# Low health literacy is associated with higher risk of type 2 diabetes: a cross-sectional study in Germany

**DOI:** 10.1186/s12889-021-10508-2

**Published:** 2021-03-16

**Authors:** Daniel Tajdar, Dagmar Lühmann, Regina Fertmann, Tim Steinberg, Hendrik van den Bussche, Martin Scherer, Ingmar Schäfer

**Affiliations:** 1 Department of Primary Care at Hamburg University Medical Center, Hamburg, Germany; 2 Hamburg Authority of Health and Consumer Protection, Hamburg, Germany

**Keywords:** Type 2 diabetes, Prevention, Diabetes risk, Health literacy, GDRS, HLS-EU-Q16

## Abstract

**Background:**

Low health literacy is believed to be associated with behaviours that increase the risk of type 2 diabetes. But there is limited knowledge on the relation between health literacy (HL) and diabetes risk, and whether improving HL could be a potential prevention strategy. Therefore, the main purpose of this study was to examine the link between HL and diabetes risk among non-diabetic adults.

**Methods:**

We used data from the Hamburg Diabetes Prevention Survey, a population-based cross-sectional study in Germany. One thousand, two hundred and fifty-five non-diabetic subjects aged 18–60 years were eligible. The German Diabetes Risk Score (GDRS, ranging 0 to 123 points) was used to determine the individual risk of type 2 diabetes. The short version of the European Health Literacy Questionnaire (HLS-EU-Q16, ranging 0 to 16 points) was applied to assess the individual self-reported HL. Subjects were asked to self-estimate their diabetes risk, which was then compared with the calculated GDRS. Descriptive statistics were calculated to investigate group differences in the GDRS and self-estimated diabetes risk. Linear as well as logistic regression models were performed to analyse potential influencing variables of the GDRS as well as incorrect self-estimated diabetes risk. In three nested statistical models for each outcome, these analyses were adjusted for age, gender, educational level and the presence of chronic conditions.

**Results:**

According to the criteria of the GDRS, 996 (79.4%) subjects showed “low risk”, 176 (14.0%) “still low risk”, 53 (4.2%) “elevated risk”, and 30 (2.4%) “high to very high risk” to develop type 2 diabetes within the next 5 years. In the statistical models including all control variables, subjects with “inadequate HL” scored 2.38 points higher on the GDRS (95% CI 0.378 to 4.336; *P* = 0.020) and had a 2.04 greater chance to estimate their diabetes risk incorrectly (OR 2.04; 95% CI 1.33 to 3.14; *P* = 0.001) compared to those with “sufficient HL”.

**Conclusion:**

The risk of type 2 diabetes is increased in people with inadequate self-reported HL. People with high diabetes risk and inadequate HL might be provided with educational programs to improve diabetes knowledge and reduce behavioural risk factors.

**Supplementary Information:**

The online version contains supplementary material available at 10.1186/s12889-021-10508-2.

## Background

About 425 million adults worldwide are estimated to have diabetes mellitus, causing total healthcare expenditures of 727 billion USD yearly [1]. Around 90% of patients with diabetes mellitus have type 2 diabetes [1]. Non-modifiable risk factors for type 2 diabetes include ethnicity, genetics and age. But there are also modifiable risk factors such as poor diet, physical inactivity and adiposity [1, 2]. It is evident that modifiable risk factors influence the development of type 2 diabetes the most, and that changes in lifestyle can reduce diabetes risk significantly [1–3].

Due to urbanization and sedentary lifestyle, the prevalence of type 2 diabetes is rising worldwide [1], and new prevention strategies are needed. One potentially modifiable risk factor for type 2 diabetes is health literacy (HL). HL is the ability of an individual to access and understand health information in order to take decisions concerning healthcare, disease prevention and health promotion [4]*.* Low HL is associated with behaviours that increase the risk of type 2 diabetes such as smoking, inactive lifestyle and poor dietary habits [5]. Prior findings suggest an inverse relationship between HL and diabetes prevalence [6, 7], and HL seems to have an impact on the health-related outcomes in patients with diabetes [5, 8–10]. Recently, results from two non-European studies indicated that HL might be associated with increased risk for developing type 2 diabetes [11, 12].

However, in Europe, every second adult has limited HL [13] and there is little knowledge on the relation between HL and the risk of type 2 diabetes. Therefore, the main purpose of this study was to examine the link between HL and diabetes risk among non-diabetic adults. In addition, we investigated the relation between HL and incorrectly self-estimated diabetes risk.

## Methods

### Study design and data sources

For this analysis we used data from the Hamburg Diabetes Prevention Survey (HDPS), a population-based cross-sectional study in Germany. The survey was conducted between December 2017 and March 2018 using computer-assisted telephone interviews.

For the HDPS, a sample of private households in Hamburg was selected by randomly dialling landline phone numbers. The selection was made according to the selection framework for telephone samples from Arbeitskreis Deutscher Markt- und Sozialforschungsinstitut (ADM) [14], a business association for German market and social research, that covers all German phone numbers. For each selected phone number that had a connection, a maximum of 10 contact attempts were made on different weekdays and at different times. The telephone interviews were conducted by USUMA (Unabhängiger Service für Umfragen, Methoden und Analysen), an independent market and social research institute [15].

A minimum sample size of 1000 randomly selected participants was defined in the study. To better represent the low socioeconomic groups, two additional waves of data collection were conducted, in which 1) a minimum of 150 subjects with no school certificate or low secondary education and 2) a minimum of 150 subjects with low or intermediate secondary education were recruited. As the HDPS was an observational study with multiple outcomes, it was not possible to carry out a sample size calculation. Instead, the sample size was determined based on the principal investigators’ experience with similar studies.

The population was selected by two screening questions at the beginning of the interviews *(“What is your highest general school leaving certificate?”* and *“What is your highest vocational qualification or university degree?”*). If the subjects from the additional waves did not meet the inclusion criteria 1) inadequately completed general education, general elementary education or basic vocational qualification or 2) intermediate qualification or A level equivalent, but no tertiary education, the interview was ended after the screening questions and the subjects were excluded from the study.

The survey based upon a standardised questionnaire including validated instruments of the 8-item Short-Form Health Survey (SF8) to measure health-related quality of life [16], the short version of the European Health Literacy Questionnaire (HLS-EU-Q16) to measure health literacy [7, 17] and items assessing sociodemographic data, health status and empowerment of the subjects. Descriptive analysis results from the HDPS were published in 2018 and 2019 [18, 19]. This manuscript was drafted following the STROBE guidelines [20].

### Study population

**Fig. 1:**
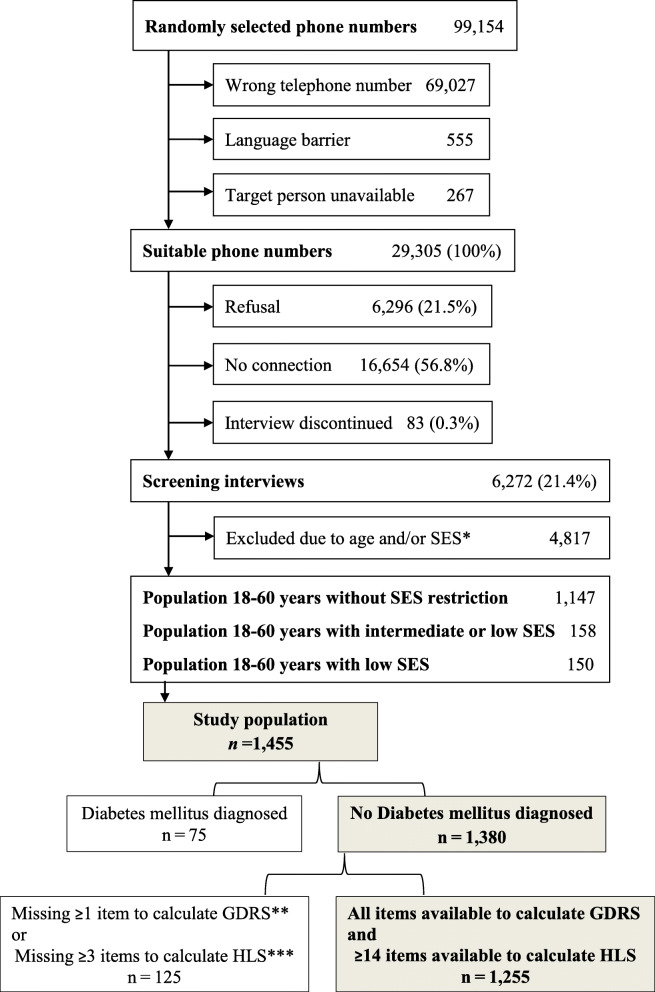
Selection of study subjects. *Socio-economic Status. **German Diabetes Risk Score. ***Health Literacy Score

For the HDPS, individuals 18–60 years old who answered the phone, and spoke German were included in the sample (Fig. 1). In households with several eligible subjects, one person was randomly selected using the “Kish selection grid” [21] based on age and gender of the household members. Non-German speaking people were excluded. For our study, we used the HDPS sample but excluded subjects who reported a diagnosis of diabetes mellitus, had ≥1 missing item in the German Diabetes Risk Score or ≥ 3 missing items in the European Health Literacy Score.

### Independent variables

Independent variables included age, gender, the presence of chronic diseases, educational level and self-reported health literacy. Age was reported in years and four age groups were categorized for the analyses: 18 to 29, 30 to 39, 40 to 49 and 50 to 60 years. Gender was reported as female or male. The presence of chronic diseases was assessed with the question “*Do you have one or more chronic diseases? Chronic diseases are long-lasting illnesses that require constant treatment and control, e.g. asthma or heart disease.*”

Educational level was assessed using the Comparative Analysis of Social Mobility in Industrial Nations (CASMIN) classification [22]. According to the criteria of CASMIN, three educational levels were categorized: Low (inadequately completed general education, general elementary education or basic vocational qualification), medium (intermediate qualification or A level equivalent) and high (lower or higher tertiary education) [22].

Self-reported HL was assessed using the HLS-EU-Q16 [17, 23], which has been applied in several studies [7, 23–26]. It includes 16 questions focusing on four HL dimensions (Table 2): accessing, understanding, appraising and applying information to take decisions concerning health care (question 1 to 7), disease prevention (question 8 to 12) and health promotion (question 13 to 16) [7, 24, 27]. Subjects were asked the questions on a four point Likert scale: Very difficult, fairly difficult, fairly easy, very easy and I do not know. The HL score was calculated, following the manual for the HLS-EU-Q16 [23]. The responses “fairly easy” and “very easy” were dichotomized to the value of 1, the responses “fairly difficult” and “very difficult” were dichotomized to the value of 0, and the response “I don’t know” was treated as missing item [23–25]. The respective values were then added to a score (minimum of 0 and maximum of 16 points) and categorized according to the criteria of the HLS-EU-Q16 as follows [23]: 0–8 “inadequate”, 9–12 “problematic” and 13–16 “sufficient” HL. The score was computed for respondents who had answered at least 14 items [7, 24].

### Outcomes

Dependent variables included a clinical assessment of diabetes risk and the self-estimated diabetes risk.

The clinical assessment of the risk of type 2 diabetes was assessed using the German Diabetes Risk Score (GDRS) [28, 29]. The score was developed by the German Institute of Human Nutrition Potsdam-Rehbruecke (DIfE) using prospective data of > 25,000 subjects and validated in other studies [30–32]. Focusing on non-invasively measured risk factors, the GDRS allows the prediction of diabetes risk within the next 5 years [30, 31]. It includes questions on age, prevalent hypertension, smoking status, family history of diabetes, body height, waist circumference, physical activity and the consumption of coffee, red meat and whole-grain bread [28, 29]. As most people do not know their individual waist circumference (wC) subjects were not asked about their wC. We calculated the estimated wC by formulas based on height, weight and age which were provided by the DIfE [28].

We categorised the GDRS (ranging 0 to 123 points) according to the recommendations of the DIfE as follows: < 46 points for “low risk” (< 2% will develop type 2 diabetes within the next 5 years), 46–56 points for “still low risk” (2 to 5%), 57–63 points for “elevated risk” (6 to 10%) and > 63 points for “high to very high risk” (> 10%) [28, 30]. Our approach based upon the original version of the GDRS without haemoglobin A1c (HbA1c) or fasting glucose test. However, it is an accurate and valid tool for prediction of diabetes risk [33].

To assess the self-estimated diabetes risk, subjects were asked to agree or disagree to the following question on a four point Likert scale: *“Compared to other people of my age and gender, I have a lower risk of developing diabetes”.* The responses “strongly agree” and “agree” were dichotomized to “yes”, the responses “disagree” and “strongly disagree” were dichotomized to “no”, and the response “I don’t know” was treated as missing value. To examine if subjects were able to estimate their diabetes risk correctly, the responses were compared with the diabetes risks resulting from the GDRS.

A correct estimation of the own diabetes risk was assumed if they answered “yes” and had a “low” or “still low” risk according to the GDRS or if they answered “no” and had a “elevated” or “high to very high” risk according to the GDRS. An underestimation was assumed if they answered “yes” and had an “elevated” or “high to very high risk” according to the GDRS. An overestimation was assumed if they answered “no” and had a “low” or “still low” risk according to the GDRS. For the analyses, underestimating or overestimating of own diabetes risk was considered as incorrect estimation. Self-estimated diabetes risk was assessed for all subjects with available data regarding these items.

### Statistical analysis

We calculated descriptive statistics for socio-demographic variables and investigated group differences in the GDRS and self-estimated diabetes risk based on the categories described above. We analysed the association between health literacy (independent variable) and the GDRS (dependent variable) by a multivariate linear regression analyses. Before analysis, we used a kernel density plot to assess normality for the diabetes risk score, which showed a slightly skewed, but approximately normal distribution for this variable (Supplementary File 1). Additionally, we analysed the association between health literacy (independent variable) and an incorrectly self-estimated diabetes risk (dependent variable) by a multivariate logistic regression analyses.

Analyses of both, GDRS and an incorrectly self-estimated diabetes risk were conducted in three nested statistical models. Model 1 was adjusted for age and gender; model 2 was adjusted for the same factors as model 1 plus the educational level; model 3 was adjusted for the same factors as model 2 plus the presence of chronic diseases. A possible improvement of the model fit by including the additional variables compared to the next variable-reduced nested model was determined by the likelihood ratio test.

Coefficients (ß) and odds ratios (OR) were reported indicating 95% confidence interval (CI). Additionally, we assessed if the interaction between health literacy and educational level had a significant association with 1) clinical diabetes risk and 2) incorrectly self-estimated diabetes risk. The level of statistical significance was set at *P* < 0.05. All tests were conducted using Stata version 15.1. Cronbach’s alpha was calculated to evaluate the internal consistency of the HLS-EU-Q16 questionnaire [34].

## Results

Initially, 1147 subjects were interviewed for the HDPS. To better represent the low socioeconomic groups, additional 150 subjects with no school certificate or low secondary education as well as 158 subjects with low or intermediate secondary education were interviewed. The HDPS sample included 1455 subjects in total.

For our study, we used the HDPS sample, but excluded 75 subjects who reported diagnosis of diabetes mellitus, and excluded 125 subjects due to missing values. Finally, 1255 subjects were eligible (Fig. 1).

### Characteristics of subjects

576 (45.9%) subjects were male and the mean age was 43.1 years (SD: 12.0). The largest age group (38.2%, *n* = 479) consisted of subjects aged 50–60 years, while subjects aged 18–29 years constituted the smallest age group (16.7%, *n* = 209) (Table 1). 212 (16.9%) subjects had low, 615 (49.0%) medium and 427 (34.1%) high educational levels. 439 (35.5%) subjects stated that they had one or more chronic diseases (Table 1).

**Table 1:** Subjects characteristics (*n* = 1255)

		n (%)
Age^a^ (years)	18–29	209 (16.7)
	30–39	236 (18.8)
	40–49	331 (26.4)
	50–60	479 (38.2)
Gender	Male	576 (45.9)
	Female	679 (54.1)
Education	Low	212 (16.9)
	Medium	615 (49.0)
	High	427 (34.1)
Presence of chronic diseases (*n* = 1237)	≥ 1 disease	439 (35.5)
	No disease	798 (64.5)
Health Literacy	Inadequate	133 (10.6)
	Problematic	414 (33.0)
	Sufficient	708 (56.4)

^a^Mean (SD): 43.1 years (12.0)

According to the criteria of HLS-EU-Q16, 133 (10.6%) subjects showed “inadequate”, 414 (33.0%) “problematic” and 708 (56.4%) “sufficient” HL (Table 1). The percentage of HLS-EU-Q16 items that were answered with *“fairly difficult”* or *“very difficult”* varied from 3.6% (*n* = 45, for question 4) to 55.6% (*n* = 698, for question 11) (Table 2). The Cronbach’s alpha for the HLS-EU-Q16 questionnaire was 0.78 [34].

**Table 2:** Percentage of HLS-EU-Q16 items for fairly difficult or very difficult (*n* = 1255) [7, 27]

*On a scale from very easy to very difficult, how easy would you say it is to: …*			*fairly difficult* or *very difficult* n (%)
No	Dimension	Subscale: Health care	
Q1	**Access**	… find information on treatments of illnesses that concern you?	253 (20.2)
Q2		… find out where to get professional help when you are ill?	230 (18.3)
Q3	**Understand**	… understand what your doctor says to you?	194 (15.5)
Q4		… understand your doctor’s or pharmacist’s instruction on how to take prescribed medicine?	45 (3.6)
Q5	**Appraise**	… judge when you may need to get a second opinion from another doctor?	429 (34.2)
Q6	**Apply**	… use information the doctor gives you to make decisions about your illness?	350 (27.9)
Q7		… follow instructions from your doctor or pharmacist?	85 (6.8)
		**Subscale: Disease prevention**	
Q8	**Access**	… find information on how to manage mental health problems like stress or depression?	462 (36.8)
Q9	**Understand**	… understand health warnings about behavior such as smoking, low physical activity and drinking too much?	52 (4.1)
Q10		… understand why you need health screenings?	78 (6.2)
Q11	**Appraise**	… judge if the information on health risks in the media is reliable?	698 (55.6)
Q12	**Apply**	… decide how you can protect yourself from illness based on information in the media?	570 (45.4)
		**Subscale: Health promotion**	
Q13	**Access**	… find out about activities that are good for your mental well-being?	301 (24.0)
Q14	**Understand**	… understand advice on health from family members or friends?	175 (13.9)
Q15		… understand information in the media on how to get healthier?	245 (19.5)
Q16	**Appraise**	… judge which everyday behavior is related to your health?	166 (13.2)

### German diabetes risk score

According to the criteria of the German Diabetes Risk Score (GDRS), 996 (79.4%) subjects had “low risk” (5 years disease probability < 2%), 176 (14.0%) “still low risk” (2 to 5%), 53 (4.2%) “elevated risk” (6 to 10%), and 30 (2.4%) “high to very high risk” (> 10%) to develop type 2 diabetes within the next 5 years (Table 3).

**Table 3:** German Diabetes Risk Score by age, gender, education, health literacy and presence of chronic diseases (*n* = 1255)

Diabetes Risk Level		low	still low	elevated	high to very high
Diabetes Risk Score (points)		< 46	46–56	57–63	> 63
5 yrs. Disease Probability^a^		< 2	2–5	6–10	> 10
		**n (%)**	**n (%)**	**n (%)**	**n (%)**
Subjects		996 (79.4)	176 (14.0)	53 (4.2)	30 (2.4)
Age (years)	18–29	200 (95.7)	8 (3.8)	0 (0.0)	1 (0.5)
	30–39	217 (92.0)	15 (6.4)	4 (1.7)	0 (0.0)
	40–49	278 (84.0)	38 (11.5)	10 (3.0)	5 (1.5)
	50–60	301 (62.8)	115 (24.0)	39 (8.1)	24 (5.0)
Gender	Male	421 (73.1)	105 (18.2)	31 (5.4)	19 (3.3)
	Female	575 (84.7)	71 (10.5)	22 (3.2)	11 (1.6)
Education	Low	147 (69.3)	40 (18.9)	12 (5.7)	13 (6.1)
	Medium	479 (77.9)	97 (15.8)	25 (4.1)	14 (2.3)
	High	369 (86.4)	39 (9.1)	16 (3.8)	3 (0.7)
Health Literacy	Inadequate	93 (69.9)	26 (19.5)	8 (6.1)	6 (4.5)
	Problematic	315 (76.1)	70 (16.9)	22 (5.3)	7 (1.7)
	Sufficient	588 (83.1)	80 (11.3)	23 (3.2)	17 (2.4)
Chronic diseases (*n* = 1237)	No disease	684 (85.7)	82 (10.3)	21 (2.6)	11 (1.4)
	≥ 1 disease	300 (68.3)	91 (20.7)	31 (7.1)	17 (3.9)

^a^*indicates how many out of 100 subjects will develop type 2 Diabetes within the next 5 years*

The diabetes risk differed between age groups (Table 3). The rate of “high to very high diabetes risk” was the highest (5%, *n* = 24) in the age group 50–60 years compared to the age groups 40–49 (1.5%, *n* = 5), 30–39 (0%, *n* = 0) or 18–29 (0.5%, *n* = 1). In all three models, increasing age was associated (*P* < 0.001) with higher risk of type 2 diabetes (Table 4). For every year, there was a 0.704 point increase in the GDRS (95% CI 0.654 to 0.754) in model 3.

**Table 4:** Potential influencing variables of the German Diabetes Risk Score: results from linear regression analyses (*n* = 1236)

	Model 1		Model 2		Model 3	
	ß (95% CI)	***p***	ß (95% CI)	***p***	ß (95% CI)	***p***
***Age (in years)***	***0.72 (0.67 to 0.77)***	***< 0.001***	***0.74 (0.69 to 0.78)***	***< 0.001***	***0.70 (0.65 to 0.75)***	***< 0.001***
***Gender: female*** **vs.** ***male***	***−6.80 (−8.03 to − 5.57)***	***< 0.001***	***−6.72 (−7.91 to − 5.53)***	***< 0.001***	***−7.09 (−8.27 to − 5.91)***	***< 0.001***
***Health Literacy:***						
- problematic vs. sufficient	1.25 (−0.09 to 2.58)	0.067	1.03 (−0.26 to 2.33)	0.117	0.53 (−0.75 to 1.81)	0.419
***- inadequate*** **vs.** ***sufficient***	***3.58 (1.52 to 5.65)***	***0.001***	***2.86 (0.86 to 4.87)***	***0.005***	***2.38 (0.38 to 4.34)***	***0.020***
***Education:***						
***- medium*** **vs.** ***low***			***−2.84 (−4.54 to − 1.14)***	***0.001***	***−2.28 (−3.96 to − 0.59)***	***0.008***
***- high*** **vs.** ***low***			***−7.68 (−9.47 to − 5.88)***	***< 0.001***	***−6.77 (−8.56 to − 4.98)***	***< 0.001***
***Presence of chronic diseases***					***4.04 (2.77 to 5.31)***	***< 0.001***

Statistically significant results (p ≤ 0.05) are shown in bolt and italic

We also observed differences in diabetes risk between women and men (Table 3). In female groups, the rate of “elevated diabetes risk” (3.2%, *n* = 22) as well as “high to very high diabetes risk” (1.6%, *n* = 11) was lower than in male groups (5.4%, *n* = 31 at “elevated risk” and 3.3%, *n* = 19 at “high to very high risk”). Female gender was consistently associated (*P* < 0.001) with lower risk for type 2 diabetes (Table 4). In women, the GDRS was 7.090 points lower than in men (95% CI − 8.270 to − 5.912).

Moreover, diabetes risk was different between educational levels (Table 3). The rate of “high to very high diabetes risk” was in the low education group (6.1%, *n* = 13) higher than in the medium (2.3%, *n* = 14) and high education group (0.7%, *n* = 3). In both respective models, education was inversely associated with the risk of type 2 diabetes (Table 4). For high education, the GDRS was 6.771 points lower than for low education (95% CI − 8.562 to − 4.980, *P* < 0.001). A 2.276 points decrease in the score was seen for medium (95% CI − 3.964 to − 0.589, *P* = 0.008) compared to low education (Table 4).

Table 3 also shows that “high to very high diabetes risk” occurred more often in people reporting at least one chronic disease (3.9%, *n* = 17) as compared to study participations reporting not to be chronically ill (1.4%, *n* = 11). People suffering from chronic diseases had a 4.041 (*P* < 0.001) point higher GDRS than people without chronic diseases. Including education and the presence of chronic conditions into our statistical models did in both cases improve the model fit (both *P* < 0.001).

**Fig. 2:**
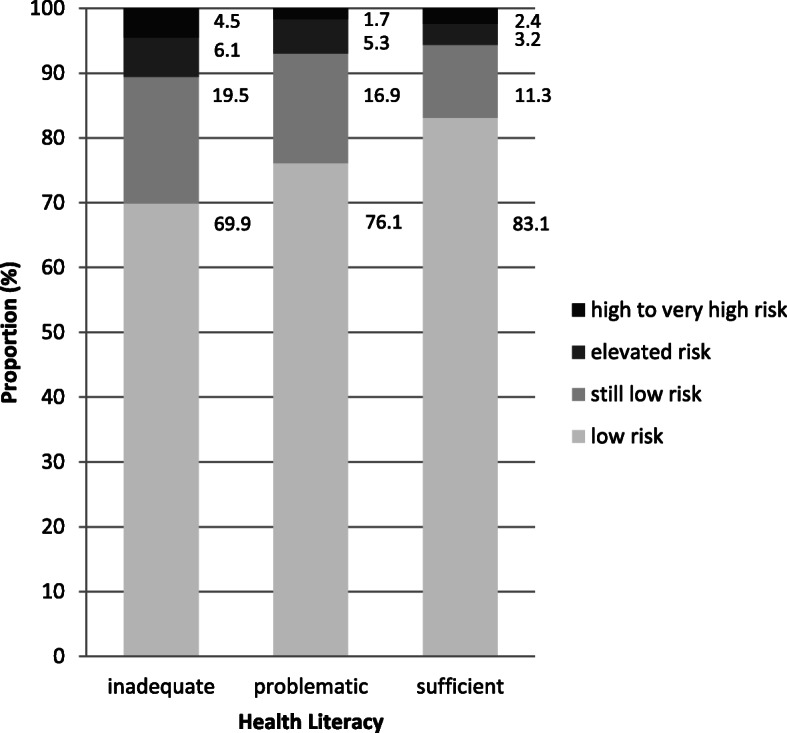
Comparison of Diabetes Risk* between Health Literacy** Levels. *categorized according to the German Diabetes Risk Score. **categorized according to the European Health Literacy Questionnaire 16

We also found differences in diabetes risk by HL level (Fig. 2). The rate of “elevated diabetes risk” increased with decreasing HL level in groups with “sufficient” HL by 3.2% (*n* = 23) in groups with “problematic” HL by 5.3% (*n* = 22) and in groups with “inadequate” HL by 6.1% (*n* = 8) (Table 3). The rate of “high to very high diabetes risk” (4.5%, *n* = 6) was in the “inadequate” HL group the highest, compared to the groups with “problematic” (1.7%, *n* = 7) or “sufficient” HL (2.4%, *n* = 17). However, in across all three multivariate statistical models, “problematic” HL did not significantly affect the GDRS (*P* = 0.419). In contrast, “inadequate” HL was significantly associated with a 2.38 points higher German Diabetes Risk Score (95% CI 0.378 to 4.336, *P* = 0.020) compared to “sufficient” HL.

We did not find an association of a) problematic (ß: -0.077; 95% CI: − 4.480 to 2.946; *p* = 0.686) and inadequate HL (ß: -0.602; 95% CI: − 5.692 to 4.488; *p* = 0.817) and medium educational level and b) problematic (ß: 1.924; 95% CI: − 1.989 to 5.837; *p* = 0.335) and inadequate HL (ß: 1.451; 95% CI: − 4.174 to 7.075; *p* = 0.613) and high educational level with the GDRS, therefore interactions were not included in the final models in Table 4.

### Self-estimated diabetes risk

One hundred forty-eight subjects (11.8%) were excluded from the analyses due to missing values. Of 1107 subjects (88.2%) with self-estimated diabetes risk, 751 (67.8%) estimated their risk correctly. Three hundred fifty-six subjects (32.2%) underestimated or overestimated their diabetes risk (Table 5).

**Table 5:** Self-estimated diabetes risk by age, gender, education, health literacy and presence of chronic diseases (*n* = 1107)

Self-estimated		Correct n (%)	Incorrect n (%)
Subjects		751 (67.8)	356 (32.2)
Age (years)	18–29	132 (68.4)	61 (31.6)
	30–39	133 (62.7)	79 (37.3)
	40–49	188 (64.8)	102 (35.2)
	50–60	298 (72.3)	114 (27.7)
Gender	Male	330 (65.6)	173 (34.4)
	Female	421 (69.7)	183 (30.3)
Education	Low	111 (62.0)	68 (38.0)
	Medium	354 (64.5)	195 (35.5)
	High	285 (75.4)	93 (24.6)
Health Literacy	Inadequate	59 (53.2)	52 (46.8)
	Problematic	231 (64.0)	130 (36.0)
	Sufficient	461 (72.6)	174 (27.4)
Chronic diseases (*n* = 1095)	No diseases	516 (73.0)	191 (27.0)
	≥ 1 disease	226 (58.3)	162 (41.8)

There was an inconsistent association between incorrectly self-estimated diabetes risk and age. In the model 1 and 2, the association was not statistically significant, but after adjusting for the presence of chronic conditions in model 3, age was significantly related to incorrect self-estimation of the diabetes risk (OR 0.87 for 10 years age difference, *P* = 0.016). In all three multivariate models, we found no association (*P* = 0.075) between incorrectly self-estimated diabetes risk and gender (Table 6).

**Table 6:** Potential influencing variables of incorrect self-estimated diabetes risk: results from logistic regression analyses (*n* = 1094)

	Model 1		Model 2		Model 3	
	ß (95% CI)	***p***	ß (95% CI)	***p***	ß (95% CI)	***p***
***Age (in years)***	−0.01 (−0.02 to 0.0006)	0.066	−0.01 (−0.02 to 0.002)	0.120	***−0.01 (−0.02 to − 0.002)***	***0.016***
Gender: female vs. male	−0.16 (− 0.42 to 0.09)	0.211	− 0.17 (− 0.43 to 0.09)	0.199	−0.24 (− 0.51 to 0.02)	0.075
***Health Literacy:***						
***- problematic*** **vs.** ***sufficient***	***0.41 (0.13 to 0.68)***	***0.004***	***0.38 (0.10 to 0.66)***	***0.007***	***0.31 (0.02 to 0.59)***	***0.033***
***- inadequate*** **vs.** ***sufficient***	***0.84 (0.43 to 1.26)***	***< 0.001***	***0.80 (0.38 to 1.22)***	***< 0.001***	***0.72 (0.29 to 1.14)***	***0.001***
***Education:***						
***- medium*** **vs.** ***high***			***0.49 (0.19 to 0.79)***	***0.001***	***0.44 (0.14 to 0.74)***	***0.004***
***- low*** **vs.** ***high***			***0.57 (0.17 to 0.96)***	***0.005***	***0.42 (0.01 to 0.82)***	***0.042***
***Presence of chronic diseases***					***0.65 (0.37 to 0.93)***	***< 0.001***

Statistically significant results (p ≤ 0.05) are shown in bolt and italic

We could see differences in self-estimated diabetes risk by educational level (Table 5). The rate of incorrectly self-estimated diabetes risk increased with decreasing educational level as follows: 24.6% (*n* = 93), 35.5% (*n* = 195), and 38.0% (*n* = 68) in groups with high, medium and low education, respectively. In our multivariate models, medium education was consistently associated with a higher chance of incorrectly self-estimated diabetes risk (OR 1.55; 95% CI 1.15 to 2.09; *P* = 0.004) compared to high education (Table 6). Odds ratios for low education (versus high education) were slightly smaller (OR 1.52; 95% CI 1.01 to 2.26) (*P* = 0.042) in model 3, but slightly larger if not controlled for chronic conditions (OR 1.76; 95% CI 1.19 to 2.61; *P* = 0.005).

Study participants reporting to be chronically ill had a higher risk for incorrectly self-estimated diabetes risk, both in descriptive analyses (41.8%, *n* = 162 vs 27.0%, *n* = 191) (Table 5) and in our multivariate model (OR 1.92; 95% CI 1.45 to 2.53; *P* < 0.001) (Table 6). The model fit was improved by both, including education (*P* = 0.002) and the presence of chronic conditions (*P* > 0.001) into our statistical models.

**Fig. 3:**
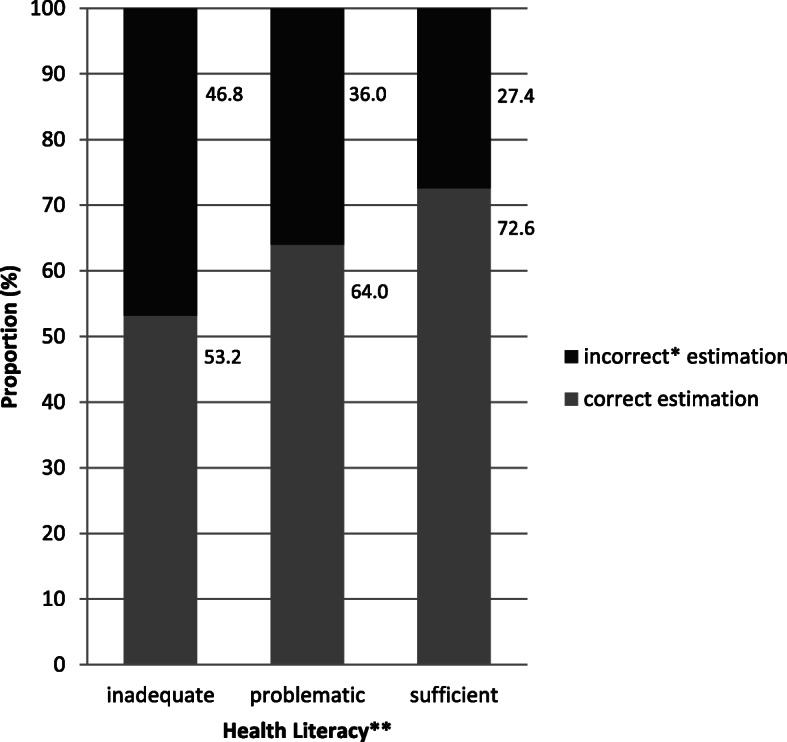
Comparison of Self-estimated Diabetes Risk between Health Literacy Levels. *underestimating or overestimating of own diabetes risk. **categorized according to the European Health Literacy Questionnaire 16

The differences between HL levels in self-estimated diabetes risk can be found in Fig. 3. The rate of incorrectly self-estimated diabetes risk increased with decreasing HL level as follows: 27.4% (*n* = 174) in groups with “sufficient” HL, 36.0% (*n* = 130) in groups with “problematic” HL and 46.8% (*n* = 52) in groups with “inadequate” HL, respectively (Table 5).

In all three multivariate models, “problematic” HL was associated with a higher chance of incorrectly self-estimated diabetes risk (OR 1.36; 95% CI 1.02 to 1.81; *P* = 0.033) compared to “sufficient” HL (Table 6). Consistently, odds ratios for “inadequate” versus “sufficient” HL were considerably higher (OR 2.04; 95% CI 1.33 to 3.14; *P* = 0.001).

There was no association of the interaction between a) problematic (ß: 0.344; 95% CI: − 0.988 to 0.299; *p* = 0.295) and inadequate HL (ß: 0.309; 95% CI: − 0.722 to 1.340; *p* = 0.557) and medium educational level and b) problematic (ß: -0.283; 95% CI: − 1.149 to 0.582; *p* = 0.521) and inadequate HL (ß: -0.185; 95% CI: − 1.420 to 1.049; *p* = 0.768) and high educational level with the incorrectly self-estimated diabetes risk. Therefore, for this outcome, there were also no interactions included in the final model in Table 6.

## Discussion

The main purpose of this study was to examine the link between health literacy (HL) and the risk of type 2 diabetes among non-diabetic adults. Our results demonstrated that the risk of type 2 diabetes is increased in people with inadequate HL, but not in subjects with problematic HL. We found that adults with problematic and inadequate HL tend to underestimate or overestimate their diabetes risk significantly. For both outcomes, we found no interaction between HL and the educational level.

Compared to a representative survey among users of the statutory health insurance in Germany [26], the rate of inadequate HL was slightly lower in our study population (10.6% versus 14.5%). This might be due to different scoring and categorisation of the HLS-EU-Q16 which we discuss in the strength and limitations section. However, the distribution of HL in our study was consistent with findings reported in the German Health Update and the European Health Literacy Survey [13, 24].

Increasing age is a non-modifiable risk factor for type 2 diabetes [1]. In line with this evidence, our results revealed a positive correlation between age and risk of type 2 diabetes.

We observed a lower risk of type 2 diabetes for women than for men. Results from six population-based studies in Germany demonstrated, that diabetes prevalence was significantly lower among women than among men [35]. Moreover, findings of a 14-year longitudinal study from Canada showed that the incidence rate of type 2 diabetes was slightly lower for women (6.3%) than for men (7.2%), but the difference was not significant [36]. However, the German Diabetes Score does not include male gender as a risk factor of diabetes [28, 29].

In our study, chronically ill subjects had a higher diabetes risk and also a higher probability for an incorrectly self-estimated diabetes risk than people who did not report to be chronically ill. A possible explanation for this finding is that many chronic diseases are either risk factors for diabetes or share the same behavioural or genetic risk factors with diabetes (e.g. cardiovascular diseases or gout) [37–39].

In our study, lower educational level was associated with higher diabetes risk among non-diabetic adults. A prevalence study from Germany revealed that the risk of having diabetes was significantly higher for low educational level compared with medium or high levels [40]. Furthermore, a case-cohort study performed in eight Western European countries found that lower educational levels were related to higher risks of type 2 diabetes incidence [41]. Correlating with a higher diabetes risk [40, 41], low education is also associated with lower HL [7, 13, 24, 42]. This leads to the assumption that diabetes risk might be higher in people with low HL. Two recent studies showed that HL was associated with increased risk for developing type 2 diabetes [11, 12]. One was from Indonesia (*n* = 42) using the Finnish Diabetes Risk Score (FINDRISC) [12] and the other one was from Australia (*n* = 1279) using the Australian Type 2 Diabetes Risk Assessment Tool [11]. To our best knowledge, our study is the first one analysing the association between HL and diabetes risk by using the German Diabetes Risk Score (GDRS) combined with the European Health Literacy Score. We found that people with inadequate HL scored on average 3.58 to 2.38 higher in the GDRS (ranging 0 to 123 points) than people with sufficient HL. At the very first glance, these additional points in the GDRS might seem little. But it has to be taken into account, that the difference between “still low diabetes risk” (46–56 points) and “high to very high diabetes risk” (> 63 points) can be just 8 points more in the GDRS.

However, there are previous studies that analysed the link between HL and diabetes prevalence [6, 7, 26]*. *Two of them suggested that lower HL was associated with higher diabetes prevalence [6, 7]*.* One was based on data of older US Americans [6] and another included an elderly East-German population [7]. However*,* the representative survey among users of the statutory health insurance in Germany did not identify such an association [26]. There are also studies that examined the relation between HL and diabetes outcomes among diabetes patients [9, 10]. Sarkar et al. found that hypoglycaemia was more common among type 2 diabetes patients that had limited HL [9]. Schillinger et al. demonstrated that among diabetes patients, inadequate HL was independently associated with worse glycaemic control and higher rates of retinopathy [10].

Our results also demonstrated that non-diabetic adults with inadequate HL had a significantly higher chance to estimate their diabetes risk incorrectly, compared to adults with adequate HL. One possible explanation might be that people with inadequate HL know less about chronic diseases than those with adequate HL as shown by other studies [8, 43, 44]. Therefore, people with low HL and high diabetes risk might benefit the most from educational programs improving their diabetes knowledge.

Overall, HL seems to have an impact on health related outcomes such as diabetes. According to the World Health Organization (WHO), HL is an important factor in preventing type 2 diabetes, as low HL is associated with modifiable risk factors such as smoking, poor diet and physical inactivity [5]. The German Diabetes Risk Score and the European Health Literacy Questionnaire can be used to select people at high diabetes risk that have inadequate HL, and help provide support through diabetes prevention programs.

In line with findings from previous publications, our results suggested a higher diabetes risk for men [35, 36, 45], increasing age [1, 29] and low education [40, 41]. Our findings also indicated that non-diabetic adults with inadequate HL have higher diabetes risk, and adults with problematic or inadequate HL tend to estimate their diabetes risk incorrectly.

### Strength and limitation

One strength of our study was the use of the German Diabetes Risk Score (GDRS) [28, 30–33] in combination with the short version of the European Health Literacy Questionnaire (HLS-EU-Q16) [7, 17, 23–25]. Both were validated and used in several studies. Including simple questions, the GDRS and the HLS-EU-Q16 can be applied and evaluated quickly by using online tools [28]. Therefore both instruments are suitable for potential diabetes prevention programs.

Comparing our results with findings from other studies could be difficult, because in recent studies two versions of HLS-EU (Q16 and Q47) were applied and differently evaluated to assess HL [7, 13, 23–26]. In some studies, HLS-EU was scored 0 to 50 points and divided in four categories (inadequate, problematic, sufficient and excellent) [7, 13, 26] while in others, it was scored 0 to 16 points and divided in three categories (inadequate, problematic and sufficient) [23–25]. We used the HLS-EU-Q16 with three categories, because it is short and easy to use [25].

Our analyses were based on a cross-sectional survey, which shows the correlation between HL and the risk of type 2 diabetes, but gives no information about the change over time and the direction of this association, e.g. if risk factors for diabetes also affect the patients’ health literacy or if health literacy has an influence on the diabetes risk. Our results should therefore be interpreted cautiously. Our data were collected using telephone interviews therefore socially-desirable answers and selection bias could be existent. The interviews were conducted in German language only. Non-German speaking adults were not interviewed which could have led to overestimation of HL in the study population [25, 46]. Also, the telephone survey was conducted in a metropolitan area only, which might have an influence on perception of medical care and – in turn – HL.

Additionally, the HDPS has a slightly uneven distribution of women (54.1%) and men (45.9%) in the sample, which probably affects the representativeness of the descriptive data. However, our multivariate data analyses identifying predictors of GDRS and incorrectly self-estimated diabetes risk have been adjusted for gender. Therefore these results should still be valid for the selected age groups in the study area.

Another limitation is the low rate of participants (21.4%, *n* = 6272) that completed the telephone interview. However, it has to be taken into account, that in our approach of generating randomized telephone numbers, many numbers presented a free line signal, but did not belong to a household. Considering this fact, our response rate would have been much higher. Furthermore increasing age is strongly associated with type 2 diabetes, thus it is most commonly seen in elderly people [1]. Aged 18–60 our study population was relatively young which could have led to underestimation of diabetes risk in general. It should also be noted that the study had to be conducted without a sample size calculation and that we therefore might have missed significant predictors of our outcomes due to limited statistical power.

## Conclusion

Our findings indicate that the risk of type 2 diabetes is increased in people with inadequate health literacy. The German Diabetes Risk Score (GDRS) and the short version of the European Health Literacy Questionnaire (HLS-EU-Q16) are practicable tools to select people at high diabetes risk that have inadequate health literacy. In order to prevent type 2 diabetes or to delay its onset, this subgroup might be provided with educational programs improving their diabetes knowledge and supporting them to change their lifestyle. Future prospective-controlled trials are needed to confirm this assumption.

## Supplementary Information

**Additional file: Supplementary File 1**. Kernel density estimate. It shows a slightly skewed, but approximately normal distribution for the diabetes risk score.

## Data Availability

The data that support the findings of this study are available from the Department of Primary Care at Hamburg University Medical Center but restrictions apply to the availability of these data, which were used under license for the current study, and so are not publicly available. Data are however available from the authors upon reasonable request and with permission of the Hamburg Authority of Health and Consumer Protection.

## Abbreviations

ADM: Arbeitskreis Deutscher Markt- und Sozialforschungsinstitut
CASMIN: Comparative Analysis of Social Mobility in Industrial Nations
DR: Diabetes risk
GDRS: German Diabetes Risk Score
HL: Health Literacy
HLS: Health Literacy Score
HLS-EU-Q: European Health Literacy Score Questionnaire
HDPS: Hamburg Diabetes Prevention Survey
USUMA: Unabhängiger Service für Umfragen, Methoden und Analysen
