# Supplementary material for: Low health literacy is associated with higher risk of type 2 diabetes: a cross-sectional study in Germany
Source: BMC Public Health. 2021 Mar 16;21:510. doi: 10.1186/s12889-021-10508-2 (PMC7962353; doi:10.1186/s12889-021-10508-2)
Supplement: Supplementary file 1 [file 12889_2021_10508_MOESM1_ESM.docx]

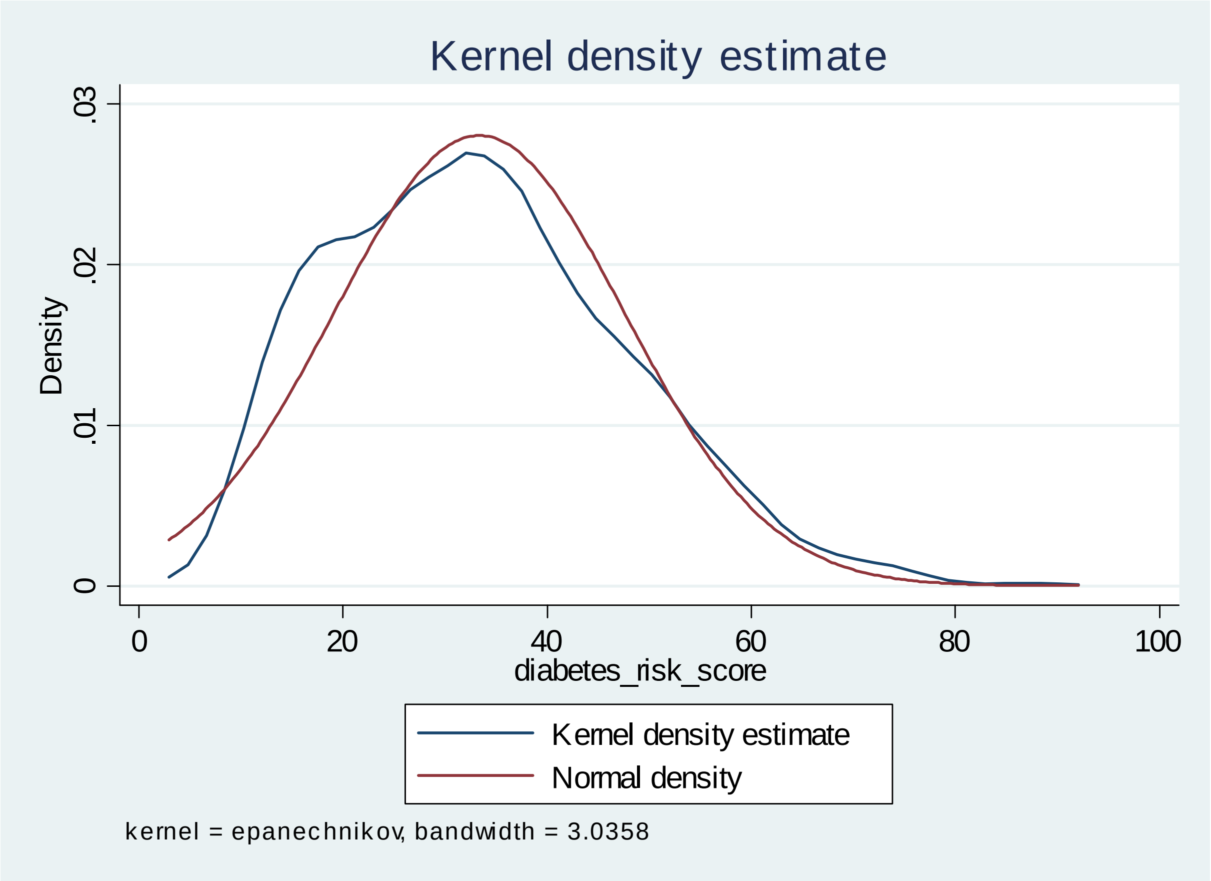
Kernel density estimate
Density
0
.01
.02
.03
0
20
40
60
80
100
diabetes_risk_score
Kernel density estimate
Normal density
kernel = epanechnikov, bandwidth = 3.0358
